# Precise nanofiltration in a fouling-resistant self-assembled membrane with water-continuous transport pathways

**DOI:** 10.1126/sciadv.aav9308

**Published:** 2019-08-09

**Authors:** Xunda Feng, Qaboos Imran, Yizhou Zhang, Lucas Sixdenier, Xinglin Lu, Gilad Kaufman, Uri Gabinet, Kohsuke Kawabata, Menachem Elimelech, Chinedum O. Osuji

**Affiliations:** 1 Center for Advanced Low-dimension Materials, State Key Laboratory for Modification of Chemical Fibers and Polymer Materials, Donghua University, Shanghai 201620, China.; 2 Department of Chemical and Environmental Engineering, Yale University, New Haven, CT 06511, USA.; 3 Department of Chemical and Biomolecular Engineering, University of Pennsylvania, Philadelphia, PA 19104, USA.; 4 ESPCI Paris, 10 Rue Vauquelin, 75005 Paris, France.; 5 Department of Chemistry, Graduate School of Science, Tohoku University, 6-3 Aoba, Aramaki, Aoba-Ku, Sendai, Japan.

## Abstract

Self-assembled materials are attractive for next-generation membranes. However, the need to align self-assembled nanostructures (e.g. cylinders, lamellae) and the narrow stability windows for ordered bicontinuous systems present serious challenges. We propose and demonstrate a novel approach that circumvents these challenges by exploiting size-selective transport in the water-continuous medium of a nanostructured polymer templated from a self-assembled lyotropic H_1_ mesophase. Optimization of the mesophase composition enables high-fidelity retention of the H_1_ structure on photoinduced cross-linking. The resulting material is a mechanically robust nanostructured polymer possessing internally and externally cross-linked nanofibrils surrounded by a continuous aqueous medium. Fabricated membranes show size selectivity at the 1- to 2-nm length scale and water permeabilities of ~10 liters m^−2^ hour^−1^ bar^−1^ μm. Moreover, the membranes display excellent antimicrobial properties due to the quaternary ammonium groups on the nanofibril surfaces. These results represent a breakthrough for the potential use of polymerized lyotropic mesophase membranes in practical water purification applications.

## Teaser

We demonstrate precise nanofiltration of molecular solutes in self-assembled membranes derived from a direct lyotropic mesophase.

## INTRODUCTION

Membrane separations are widely used in existing technological applications including seawater desalination, gas separation, food processing, and fuel cells, as well as in emerging areas, such as sustainable power generation and distillation (*1*). Nanofiltration involves the removal of dissolved or suspended solutes ranging from 1 to 10 nm in size. The development of new nanofiltration membranes is of particular interest for low-cost treatment of wastewaters to remove organic contaminants, including so-called contaminants of emerging concern such as pesticides and metabolites of pharmaceutical drugs (*2*). Current state-of-the-art membranes, however, suffer from a generally recognized trade-off between permeability and selectivity: Increasing permeability often results in decreased selectivity and vice versa (*3*, *4*). This trade-off originates from the intrinsic structural limitation of these conventional membranes, i.e., a broad distribution of free volume elements in dense polymer membranes or pore sizes in porous membranes. Membranes based on self-assembled materials entail the use of nanostructures with near-monodisperse critical dimensions. Self-assembled materials have therefore been considered an attractive way to realize highly selective separations without compromising permeability (*5*–*7*).

**Fig. 1:**
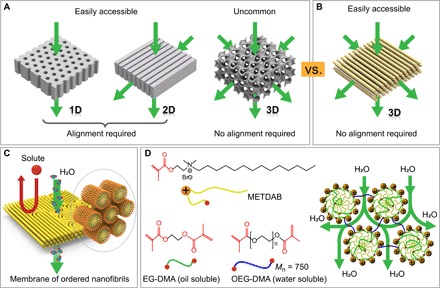
Schematic illustration of self-assembled structures used to fabricate nanoporous polymer membranes. (**A**) The two readily obtained morphologies, i.e., lamellae and cylinders, require alignment of the structures to optimize transport. (A) Schematic illustration showing self-assembled morphologies used as templates for fabricating nanoporous polymer membranes. Two easily obtained morphologies, i.e., lamellae and cylinders, while used for forming nanopores, require alignment of the self-assembled domains. 3D interconnected gyroids are not universally observed in BCP and LC systems and, where they occur, usually exhibit narrow windows of stability. (**B**) Proposed morphology for fabricating membranes that can be easily templated from mesophases of hexagonally packed cylinders and requires no alignment to enhance flux. (**C**) Schematic illustration for preparation of size exclusion nanofiltration membranes from cross-linking of a direct hexagonal cylinder lyotropic mesophase (H_1_). The cross-linked sample contains hexagonally packed molecular fibrils in the continuous water phase, which allows water to permeate through the gap between nanofibers but rejects larger-size solutes because of size exclusion. (**D**) Molecular structures of the polymerizable surfactant METDAB, the water-soluble cross-linker OEG-DMA, and the oily cross-linker EG-DMA for the formulation of the desired H_1_ mesophase. EG-DMA (in green) copolymerizes with the surfactant in the hydrophobic core, and water-soluble OEG-DMA (in blue) bridges each cylinder into a network, the morphology of which provides a continuous aqueous transport path, as schematically illustrated. *M*_n_, number-average molecular weight.

Block copolymers (BCPs) and small-molecule liquid crystals (LCs) can self-assemble into a series of mesophase morphologies having periodic nanoscale domains with sizes and shapes that are thermodynamically defined. The well-ordered nanostructures found in BCPs and LCs, including cylinders (*8*, *9*), lamellae (*10*), and gyroids, have been considered as attractive templates for the fabrication of nanoporous membranes (*8*). Uniform-size nanopores may already exist intrinsically in some self-assembled systems, or they can be formed by selective removal of a sacrificial component (*11*–*13*). Membranes made by non–solvent-induced phase separation of BCPs represent a compelling advance in terms of selectivity (at the ~10-nm length scale) and scalability of fabrication (*14*–*17*). In addition, self-assembling materials provide useful templates for controlling the organization of discrete objects such as water channel proteins (*18*) or nanotubes (*19*), which can function as nanofiltration pores. While these nanoporous membranes have been demonstrated to show high selectivity and permeability for nanofiltration and ultrafiltration, challenges are still encountered in their practical development, as schematically outlined in Fig. 1A.

For the readily accessed one-dimensional (1D) cylindrical and 2D lamellar systems, the anisotropic nature of the nanostructures implies the need for uniform orientation throughout the system, e.g., vertical cylinders in a thin film, to produce an optimized morphology for membrane performance. These optimized morphologies, however, do not spontaneously occur during membrane fabrication processes. Considerable efforts must be made to direct the self-assembly of nanostructured domains in thin films. To this end, previous studies have successfully used interfacial engineering, e.g., top coating and surface anchoring (*20*–*22*), or external fields such as magnetic fields (*11*, *23*, *24*). Although these methods are reliable, they bring additional constraints that present considerable, if not insurmountable, challenges for large-scale membrane fabrication. On the other hand, 3D interconnected gyroid nanopores are advantageous over their cylindrical or lamellar counterparts, because the nanopores need no alignment to ensure continuity and optimized permeability in the resulting membrane (*25*–*27*). However, access to gyroid morphologies is complicated by their generally narrow windows for phase stability in BCPs (*28*) and by the tailored molecular structures required in LC assemblies (*29*, *30*). The aforementioned challenges have combined to hamper the pursuit of high-performance membranes derived from self-assembled materials. In addition to the need to optimize transport morphology, biofouling resistance is a major concern. The generally poor biofouling resistance of current water treatment membranes and difficulties associated with their cleaning increase operating costs and are an important challenge to overcome (*31*, *32*).

Here, we report a scalable approach to obtain highly permeable and selective nanofiltration membranes that also exhibit attractive anti-biofouling properties, specifically antimicrobial activity. The membranes take advantage of a morphology consisting of hexagonally ordered molecular nanofibrils (Fig. 1B). This morphology is realized by cross-linking a direct cylindrical (H_1_) lyotropic LC (Fig. 1C). In contrast to gyroid phases, the H_1_ mesophase occurs more frequently and exhibits stability in a much wider composition window in lyotropic systems. While the H_1_ phase obviates the need for alignment, care must be taken in the formulation of the system to ensure the ability to cross-link in place, without loss of structure. The membranes described here are based on mesophases that have been optimized for high-fidelity retention of the structure of the lyotropic precursor in the cross-linked system. The membrane is mechanically robust and is resilient against both dehydration and swelling by excess water. We surmise that these properties originate because of structural cohesion provided by topological defects present in the system and water-bridging cross-links between cylindrical structures. The notion that a polymerized direct lyotropic system would retain its structure in aqueous media is counterintuitive and represents a strong departure from previous reported work (*33*). The self-assembled structure provides a uniform and well-defined spacing between nanofibrils, thereby leading to high membrane selectivity. The availability of a 3D-continuous transport path in the membrane obviates any need for structural alignment, thereby significantly reducing the complexity of membrane fabrication. These characteristics definitively set the currently reported membranes apart from nanostructured membranes derived from lyotropic LCs reported to date and offer a path toward viability in practical nanofiltration operations.

## RESULTS AND DISCUSSION

A cationic surfactant, 2-(methacryloyloxy)ethyl tetradecyl dimethyl ammonium bromide (METDAB), bearing a polymerizable methacrylate group close to the hydrophilic head, was used to formulate a polymerizable H_1_ mesophase with water and additional cross-linkers, ethylene glycol dimethacrylate (EG-DMA) and oligo(ethylene glycol) dimethacrylate (OEG-DMA) (Fig. 1D). The surfactant monomer, or surfmer, was synthesized in a single-step Menshutkin reaction (*34*), details of which can be found in Materials and Methods. The METDAB/water binary phase diagram at room temperature displays isotropic micellar solution (L_1_), hexagonal cylindrical phase (H_1_), bicontinuous gyroid phase (G), and lamellar phase (L_α_), as the surfactant concentration is increased from 0 to 100 weight % (wt %) (fig. S1). The formation of H_1_ phases at room temperature occurs in a range of METDAB concentration from roughly 55 to 80 wt %.

One approach to obtain an ordered polymerized nanofibril structure is by photoinitiated polymerization of H_1_ mesophases (*35*). For membrane applications, it is of critical importance to achieve high-fidelity replication of the ordered nanostructures from the LC template. Previous studies have suggested the utilization of cross-linking to retain LC order, by introducing multiple polymerizable groups to the reactive amphiphiles (*25*, *30*) or by adding cross-linkers into the systems (*10*, *20*, *36*, *37*). For the polymerization of H_1_ mesophases formed by reactive surfactants bearing single polymerizable groups, hydrophobic cross-linkers that segregate into the micellar cores have been commonly used (*36*, *37*). While success has been claimed in some cases, previous studies on polymerization of H_1_ systems have typically relied on qualitative interpretations provided by 1D x-ray diffraction data and low-resolution polarized optical microscopy (POM) images for structural characterization. Missing in these efforts has been high-resolution real-space imaging, for example, by transmission electron microscopy (TEM) or atomic force microscopy (AFM), of the nanostructures produced after cross-linking. This real-space imaging along with higher-resolution POM characterization is vitally important for assessing the fidelity of structure retention after cross-linking. X-ray scattering data alone are unfortunately often less sensitive than required. It is therefore unclear to what extent structural preservation is achieved in the reported systems. Here, we performed detailed structural characterizations using a combination of high-resolution small-angle x-ray scattering (SAXS) with high-resolution microscopy (high-resolution POM and direct imaging by TEM and AFM) to verify the retention of our formulated H_1_ mesophase after ultraviolet (UV)–initiated cross-linking.

Results of cross-linking experiments carried out on H_1_ mesophases that are not optimally formulated highlight the points made above regarding structure retention and appropriate characterization thereof. Photoinitiated polymerization of H_1_ mesophases simply formed by METDAB/water binary systems in a broad range of compositions resulted in substantial disruption of the H_1_ morphology, as evidenced by the apparent cloudiness developed in the polymerized samples, the indiscernible LC textures in POM images, and the shift of the SAXS peak ratio from 1:$\sqrt{3}$ to 1:$\sqrt{4}$ (fig. S2). Although the presence of an added cross-linker may improve the structural retention of the system on polymerization, disruption still occurs. While the disruption is not easily recognized at first by x-ray scattering because of the preservation of the 1:$\sqrt{3}$ SAXS peak ratios and POM, it is seen in the subtle changes to the LC texture of developable domains in high-resolution POM images and the tendency to form lamellar structures observed in TEM images that may explain the unexpected increase of the (200) peak intensity after polymerization found in both the system here and a previous study (fig. S3) (*37*). Our observations mirror the polymerization-induced phase transformation or phase separation observed in lyotropic mesophases (*38*).

**Fig. 2:**
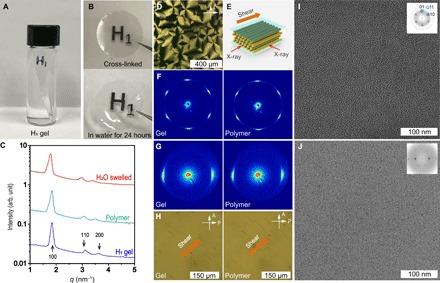
High-fidelity retention of the H_1_ mesophase morphology after UV-induced cross-linking with the aid of dual cross-linkers. (**A**) Photo of an H_1_ mesophase gel formed by 70 wt % METDAB, 22.8 wt % water, 5.4 wt % OEG-DMA, and 1.8 wt % EG-DMA. (**B**) Photos showing the corresponding cross-linked polymer film (40 μm thick) and the film integrity after immersion in water for 24 hours. (**C**) 1D integrated SAXS data display the structural consistency of the H_1_ morphology in the non–cross-linked gel, the cross-linked polymer, and the polymer after immersion in water for 24 hours. A small increase of the *d*_100_ spacing from 3.6 to 3.7 nm was found after 24 hours of water immersion, indicating that there was very little swelling of the sample. (**D**) POM image displaying the preservation of the typical LC texture found in cylindrical mesophases. (**E**) Schematic illustration of the shear alignment and the 2D SAXS measurements. 2D SAXS patterns before and after cross-linking, as obtained by incidence of the x-ray beam (**F**) parallel and (**G**) orthogonal to the shear direction. (**H**) POM images showing the essentially unchanged birefringent color of the oriented cylindrical micelles before and after cross-linking. The sample was positioned such that the original shear direction was at 45° with respect to each of the two crossed polarizers. TEM micrographs viewed along (**I**) and orthogonal to (**J**) the shear direction showing aligned nanofibrils. Insets: Fast Fourier transform (FFT) images. Before microtoming, the polymer was immersed into a 0.1 wt % KI aqueous solution for 1 hour to replace Br^−^ by I^−^ to enhance the atomic number contrast for imaging. Photo credit: Xunda Feng, Yale University.

We used a dual cross-linking strategy to more robustly preserve the H_1_ morphology and to thereby circumvent the structural disruption issue described above: EG-DMA is insoluble in water and therefore presumably sequesters selectively within the hydrophobic cores of the cylindrical micelles of METDAB. Addition of EG-DMA is therefore expected to help cross-link the interior of the cylindrical micelles into nanofibrils. Conversely, we pursued the addition of a hydrophilic counterpart, OEG-DMA, with the express intention that it bridges the aqueous spaces between the cross-linked nanofibrils to form a tight network (Fig. 1D). In this perspective, OEG-DMA serves not only to improve morphology retention during polymerization but also to provide mechanical integrity in the resulting polymer films, which must be robust enough to permit pressure-driven permeation in use as membranes. An optimized composition of 70 wt % METDAB, 22.8 wt % water, 5.4 wt % OEG-DMA, and 1.8 wt % EG-DMA was developed that formed a stable, homogeneous H_1_ mesophase gel (Fig. 2A). The EG-DMA was itself mixed with a small amount of the photoinitiator 2-methoxy-2-phenylacetophenone (10 wt %) to facilitate photoinduced cross-linking of the mesophase. The photoinitiator concentration in the system overall was therefore 0.18 wt %.

Phase transformation and phase separation on UV-induced cross-linking were successfully suppressed for the optimal gel composition, as reflected by the excellent transparency of a representative resulting sample (Fig. 2B). Moreover, the observed physical integrity on handling and the preservation of high optical transparency of the film after immersion in water for 24 hours indicate that the system is resistant against structural collapse due to water swelling. This retention of structural integrity is a critical necessity for a working membrane that remains in contact with aqueous streams for extended durations. Rheological measurements indicate that the shear modulus of cross-linked H_1_ films is approximately 0.1 GPa, highlighting the mechanical integrity of the materials.

SAXS data provide more reliable information on the structural retention after cross-linking and subsequent water swelling (Fig. 2C). The unchanged ratio of scattering peak locations (1:$\sqrt{3}$:$\sqrt{4}$) as shown in the 1D integrated data demonstrates the intact hexagonal morphology after cross-linking. The *d*_100_ spacing of 3.6 nm was the same before and after UV exposure, highlighting the fact that there was no change of lattice parameter during cross-linking. The primary Bragg peak width became marginally broader after cross-linking, with an increase of 0.018 nm^−1^ in the full width at half maximum (fig. S4), suggesting a slight reduction in structural correlation length after polymerization. As for the polymer immersed in water for 24 hours, we observed negligible swelling of the network, evidenced by a small (~2%) increase of the *d*_100_ spacing from 3.6 to 3.7 nm. These findings demonstrate the effectiveness of our dual cross-linker strategy for retaining structural order after cross-linking and producing a mechanically resilient nanostructured film with water-continuous domains. Time-dependent SAXS measurements on the influence of the *d*_100_ spacing by water swelling suggest that the ~2% lattice parameter change occurred within 30 min (fig. S4). In addition, the structural robustness of the cross-linked film after swelling is further reflected in the maintenance of the developable domain LC texture shown in the POM image (Fig. 2D). Furthermore, larger-area and higher-resolution views in POM images also display no optical inhomogeneity within the LC domains (fig. S4).

The retention of the original H_1_ structure in the cross-linked polymer is also apparent by comparing SAXS data of a shear-aligned specimen before and after UV exposure. Figure 2E shows the schematic illustration of the shear-induced alignment and the incident directions of the x-ray on the sample. 2D SAXS images as obtained by incidence of the x-ray beam along and orthogonal to the shear direction display patterns with six- and twofold symmetries, respectively, which can be preserved in the corresponding cross-linked polymer (Fig. 2, F and G). The unperturbed orientations of both cylindrical axes and hexagonal lattices strongly demonstrate that the H_1_ morphology was effectively locked in by cross-linking. As expected, POM images further show the same birefringent color of the sheared aligned sample before and after cross-linking (Fig. 2H).

A high-resolution TEM image, as shown Fig. 2I, was obtained for an approximately 150-nm-thick section microtomed perpendicular to the shear direction (fig. S5). The polymer sample before sectioning was stained by immersing into a 0.1 wt % KI aqueous solution to enhance atomic number contrast. An ordered array of hexagonally packed nanofibrils can be observed, with the inset fast Fourier transform (FFT) pattern displaying sixfold symmetry. The cores of the nanofibrils shown in the TEM are brighter than the matrix because of the reduced electron transparency of the fibril outer wall, as stained by iodine ions. A *d*_100_ spacing of 3.6 nm was calculated from the FFT pattern of this TEM image, in good agreement with the SAXS data. TEM visualization orthogonal to the shear direction (Fig. 2J) displays the orientation of the nanofibrils along the shear direction and the corresponding FFT image with twofold symmetry. To the best of our knowledge, these are the first TEM data that show the successful production of ordered nanofibril arrays from polymerization or cross-linking of lyotropic H_1_ mesophases. On the basis of the structural characterization data, we anticipate controlling dimensions of ~1.1 and ~0.5 nm for transport parallel and perpendicular to the nanofibrils, respectively (fig. S6).

**Fig. 3:**
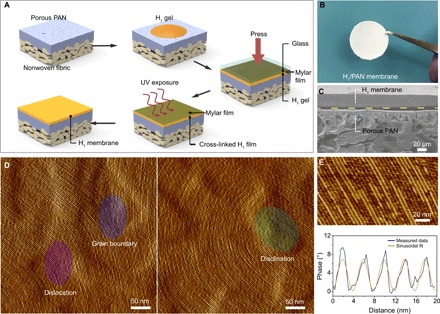
Fabrication of H_1_/PAN composite membranes and microscopic characterization of the membrane structures. (**A**) Schematic illustrating H_1_ membrane fabrication on supporting PAN membranes. (**B**) Photograph of H_1_/PAN composite membrane. (**C**) Cross-sectional SEM image showing the composite membrane. (**D**) AFM images showing the surface morphology of the H_1_ membrane with closely packed nanofibrils. Topological defects including dislocations, disclinations, and grain boundaries are well preserved on the film surface, consistent with high-fidelity retention of the mesophase morphology on cross-linking. (**E**) Line profile analysis of the high-resolution image (scale bar, 20 nm) shows an interfibril distance of ~4 nm, in good agreement with SAXS and TEM measurements. Sinusoidal fit of the line profile provides a guide to the eye. Photo credit: Xunda Feng, Yale University.

The water-continuous nature, mechanical resilience, and ordered self-assembled morphology of the cross-linked H_1_ mesophase are attractive for membrane applications. The ability of the system to function as a membrane was assessed in a proof-of-concept manner. Membranes were produced by cross-linking a thin film of the H_1_ mesophase spread on commercially sourced polyacrylonitrile (PAN) ultrafiltration membranes (with molecular weight cutoff of ~400 kDa; Sterlitech) that were used as mechanical supports. As schematically illustrated in Fig. 3A, the process involves spreading of the H_1_ gel onto a PAN support, followed by a mechanical pressing step to produce a thin gel film, and subsequent UV exposure for cross-linking (detailed procedures in Materials and Methods). The resulting cross-linked H_1_ film was contiguous with the supporting PAN membrane, as shown in the photo of the H_1_/PAN composite membrane (Fig. 3B) and the scanning electron microscopy (SEM) image of the cross-sectional view (Fig. 3C). The continuity of structure (i.e., absence of delamination) suggests that there is appreciable adhesion between the film and the PAN. The mesophase typically penetrated some distance (about tens of micrometers) into the PAN support during pressing (fig. S7). While this penetration could be useful in avoiding delamination, it lends uncertainty in the determination of the effective thickness of the H_1_ film. In terms of the well-identified layer above the PAN surface, the procedure resulted in H_1_ films with thickness in the range of approximately 3 to 30 μm, as visualized by SEM.

The surface morphology of the H_1_ membrane was characterized by AFM. Planarly oriented arrays of nanofibrils connected by typical topological defects including grain boundaries, dislocations, and disclinations can be clearly observed in the high-resolution AFM images (Fig. 3D). An interfibril spacing of approximately 4 nm was obtained from the cross-sectional analysis of an AFM image (Fig. 3E). The morphology of randomly oriented nanofibril domains may contribute to the membrane’s structural integrity because of the effective entanglement provided by topological defects connecting domains of different orientation. However, given the structural stability observed on extended swelling studies as well as on drying, the role of OEG-DMA in covalently cross-linking nanofibrils is expected to be equally, if not more, important.

**Fig. 4:**
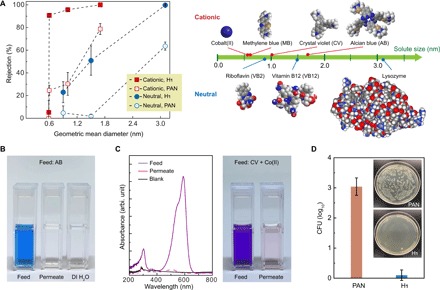
Solute rejection and antibacterial properties of H_1_ membranes. (**A**) Rejection data of H_1_ membranes and PAN membranes for seven different solutes: Co(II), MB, CV, AB, VB2, VB12, and lysozyme. Spacing filling models and estimated geometric mean diameters of the solutes are shown. Error bars represent 95% *t* test confidence limits derived from data variance across multiple measurements, typically two membranes and four permeate samples per membrane. (**B**) Photos of the feed solution of AB, the permeate from the H_1_ membrane, and deionized (DI) water as a reference, respectively. (**C**) UV-visible (UV-Vis) spectrum and photo showing competitive solute separation of CV and Co(II). (**D**) Quantification of bacterial growth in colony-forming units (CFU) in the samples and photographs (insets) showing control and H_1_-derived membrane samples after incubation with bacteria. Photo credit: Yizhou Zhang, University of Pennsylvania and Xinglin Lu, Yale University.

Solute rejection experiments were assessed by challenging the H_1_ composite membrane with solutions containing a series of charged (cationic) and neutral molecules, with geometric mean sizes (diameters) ranging from 0.6 to 3.1 nm in a pressurized stirred cell (detailed in fig. S8). Anionic dyes were not used to avoid the potentially confounding role of molecular fouling due to adsorption onto the positively charged nanofibril exterior. Experiments were conducted over extended periods (several hours to several days) to ensure that the results were representative of steady-state performance rather than reflecting any transient effects due to dead space in the filtration cell, solute adsorption, membrane compaction, or any inadvertent leaching of material from the system (fig. S9). The results of single-solute rejection experiments are summarized in Fig. 4A. The solute sizes are geometric mean sizes determined from molecular dimensions calculated using the Chem3D software package. The supporting PAN membrane data are given as the control. The composite membrane displays strong size selectivity toward charged organic dye molecules, demonstrating rejection of ~90% or higher for methylene blue (MB; ~320 Da), crystal violet (CV; ~408 Da), and Alcian blue 8G (AB; ~1300 Da). Complete AB rejection by the H_1_ membrane is visible from the colors of the feed solution and the permeate from the H_1_ membrane, respectively: The blue color from AB effectively disappears from the permeate (Fig. 4B). Meanwhile, the lower solute rejection of the PAN support by itself indicates that the selectivity of the composite membrane is dominated by the H_1_ layer. The H_1_ membrane did not reject CoCl_2_, suggesting that the effective pore size in the system is larger than the limiting 0.8 nm diameter of hydrated Co^2+^ ions. The molecular weight cutoff and size cutoff for the charged solutes were ~350 Da and 1 nm, respectively. The membranes were also selective against neutral solutes but with a shift in the cutoffs to ~4 kDa and ~2.5 nm. The H_1_ composite membrane completely rejects lysozyme (~14.3 kDa) at the isoelectric point, while both cobalamin (VB12; ~1400 Da) and riboflavin (VB2; ~380 Da) are only moderately rejected.

The transport data indicate that the membranes can separate solutes effectively on the basis of size as well as charge. Competitive rejection tests involving filtration of solutions containing two solutes were performed, for solutes of different size and different charge, respectively. In the former case, a mixture of CoCl_2_ and CV was fully separated, with complete rejection of CV and zero rejection of Co^2+^ (Fig. 4C). For the latter case, the membrane was challenged with a mixture of CV and VB2. Analysis of the permeate shows that CV was completely rejected by the membrane, while the VB2 was approximately 50% rejected (fig. S10).

The rejection data highlight the important role of electrostatic interactions, i.e., Donnan exclusion, in the transport properties of these membranes. At the same time, relative to the theoretical transport dimensions of the mesophase (section S1) and under the assumption that diffusion through the nanofibrils (rather than flow around them) is prohibited, the rejection data for neutral solutes suggest that transport may be compromised by the presence of defects of some sort. It is possible that these defects originate because of imperfections associated with the mechanical pressing or another step in the membrane fabrication process. Another possibility is that topological defects in the mesophase provide less restrictive paths for solute transport. It is likely that continued refinement of the membrane fabrication process, and specifically a departure from mechanical pressing methods, will improve the selectivity of the membranes by reducing defects such as those described here. Note, however, that the assumption of zero permeability through the nanofibrils has not been rigorously tested here. While we expect that diffusion of water-soluble dyes through the hydrophobic nanofibrils would represent a very high resistance pathway, in the limit of subnanometer-scale interstitial spaces, it represents the only transport pathway for solutes. Additional investigation along these lines may be fruitful, particularly in terms of highlighting the boundary between porous and solution diffusion mechanisms in polymer membranes.

The thickness-normalized pure water permeability of the H_1_ membranes was determined to be ~10 liters m^−2^ hour^−1^ bar^−1^ μm, using free-standing membranes to avoid the uncertainty in thickness due to PAN penetration. Water permeability decreased by up to 50% during filtration of charged solutes but was unaltered in the presence of neutral solutes. The high water fluxes observed for the system are consistent with the presence of a physically continuous transport path. Notably, the water permeability is considerably higher than that reported for membranes templated from gyroid LCs (*25*). It is anticipated that a high water flux well above 100 liters m^−2^ hour^−1^ bar^−1^ can be realized when the thickness of the H_1_ film is reduced to the range of 200 nm. These permeabilities, current and anticipated, compare favorably to those of commercial nanofiltration membranes, such as Dow FILMTEC NF90-400 that have permeabilities of roughly 10 to 15 liters m^−2^ hour^−1^ bar^−1^.

The presence of the water-facing quaternary ammonium groups on the nanofibrils due to the surfactant self-assembly suggests that the membranes may exhibit anti-biofouling behavior because of the well-established antimicrobial properties of these functional groups (*39*, *40*). The potential for anti-biofouling behavior of H_1_ membranes was studied using a standard colony-forming unit (CFU) enumeration assay (details in the Materials and Methods) (*41*, *42*). Bare PAN membranes were also investigated as a control for comparison. In a typical experiment, a PAN or an H_1_ membrane was kept in contact with a model Gram-negative bacterium (*Escherichia coli*) in suspension for 3 hours. The membranes were mildly sonicated in saline solution to detach bacteria from their surfaces that were subsequently cultured on agar and incubated overnight. Photos in Fig. 4D show the agar plates of *E. coli* colonies cultured from cells on the control and the H_1_ membrane. The CFU data show that the number of viable *E. coli* cells from the H_1_ membrane was three orders of magnitude smaller than that of the control. The strong reduction in CFU for H_1_ membranes is consistent with a strong antimicrobial response, as anticipated because of the presence of the quaternary ammonium groups. Note, however, that biofouling due to bacterial growth is only one aspect of the complex problem of membrane biofouling and fouling overall.

## CONCLUSION

In conclusion, we have reported here a facile approach to fabrication of polymer nanofiltration membranes with a unique morphology of ordered arrays of nanofibrils. The approach uses a cross-linkable, water-continuous lyotropic H_1_ mesophase as a template to realize the desired morphology. Our formulation of the H_1_ mesophase takes the advantage of dual cross-linkers to preserve the ordered nanostructures with high fidelity and ensure mechanical robustness of the resulting membrane. Systematic structural characterizations using POM, SAXS, high-resolution AFM, and TEM unambiguously confirm the formation of highly ordered nanofibrils in the cross-linked polymer membranes. Moreover, the direct imaging by TEM and AFM represents, to the best of our knowledge, the first such characterization of a polymerized lyotropic H_1_ mesophase. The main constituent species of the system, METDAB, can be synthesized in large quantities in a single step using readily available and inexpensive reagents. Production of large-area, highly permeable, and molecularly selective membranes for nanofiltration involves simple photoinduced cross-linking of the mesophase without any alignment procedure.

The membranes exhibit clear size-based selectivity when challenged with molecular dyes as model solutes and demonstrate thickness-normalized water permeabilities of ~10 liters m^−2^ hour^−1^ bar^−1^ μm. This high water flux is linked to the water-continuous structure templated from the water-continuous lyotropic LC. The disparity in the molecular weight cutoff for passage of neutral solutes relative to the theoretical limiting dimensions of the mesophase suggests that defects are present. A reduction of these defects, for example, by improvements in fabrication processes, can improve membrane selectivity. In the limit of very small interstitial spaces and low defect densities, these membranes may enable an assessment of the crossover from porous transport to solution diffusion.

We anticipate that additional improvements can be obtained by optimizing fabrication procedures to provide thinner selective layers, as well as through modification of the surface chemistry of the nanofibrils by the use of cosurfactants or postfunctionalization to tune the cutoff characteristics of the membrane. Last, the derived membranes demonstrate excellent antimicrobial activity due to the intrinsic presence of quaternary ammonium groups. This antimicrobial activity is beneficial in mitigating biofouling, which is a pressing concern in practical membrane applications.

## MATERIALS AND METHODS

### Materials

All chemicals used in this study were purchased from Sigma-Aldrich and used as received unless otherwise noted. The water-soluble cross-linker OEG-DMA has an average *M*_n_ (number-average molecular weight) of 750, as specified by the supplier. The radical photoinitiator 2-methoxy-2-phenylacetophenone was dissolved into the oil-soluble cross-linker EG-DMA at a concentration of 10 wt %.

### Synthesis of polymerizable surfactant METDAB

The polymerizable surfactant METDAB was synthesized by one-step Menshutkin reaction using a slightly varied procedure, as reported in the literature (*34*). 2-(Dimethylamino)ethyl methacrylate (31.4 g, 0.2 mol), 1-bromotetradecane (55.4 g, 0.2 mol), and anhydrous acetone (100 g) were mixed in a round-bottom flask. The mixture was stirred and heated at 45°C in an oil bath for 48 hours. After the reaction, the product, a white solid, was precipitated from the solution by adding an excess amount of diethyl ether to the flask and then filtered using a Büchner funnel. The crude product was purified by recrystallization in ethyl acetate. The final product was rinsed several times using diethyl ether and filtered, followed by drying in vacuum for 12 hours before use. The yield was above 70%.

### Formulation of lyotropic LCs and cross-linking

Lyotropic LCs can be formed by simply mixing the polymerizable surfactant METDAB and water. To preserve the mesophase morphologies, additional cross-linkers may be introduced into the system. The binary surfactant/water phase diagram was obtained by systematic variation of the weight ratio of surfactant to water and characterization of the corresponding surfactant/water mixtures using POM and x-ray scattering. The formation of direct hexagonal phases (H_1_) at room temperature was found to locate in the window of METDAB contents from 55 to 80 wt %. The cross-linkable H_1_ phase used for the preparation of H_1_ polymer membranes was obtained by mixing 70 wt % METDAB, 22.8 wt % water, 5.4 wt % OEG-DMA, and 1.8 wt % EG-DMA (doped with a radical initiator). This formulation was able to be cross-linked and to give the excellent preservation of the H_1_ morphology. Cross-linking/polymerization of mesophases was conducted in an N_2_ atmosphere using a focused spot UV beam for 30 min (100-W Sunspot SM spot curing system at a distance of ∼2 cm).

### Polarizing optical microscopy

POM studies on LC textures were performed using a Zeiss Axiovert 200 M inverted microscope. LC samples sandwiched by two glass slides were slightly heated to facilitate the formation of the typical LC texture before POM visualization.

### X-ray scattering

2D x-ray scattering data of mesophases before and after cross-linking were obtained using a Rigaku S-MAX3000 instrument with the accessible scattering vector (*q*) ranging from 0.015 to 0.24 Å^−1^. The wavelength of the x-ray beam was 1.542 Å (Cu Kα radiation). X-ray scattering with higher *q* values was performed using a Rigaku MicroMax 007 HF+ instrument with a rotating anode Cu Kα x-ray source and a 2D Saturn 994+ charge-coupled device detector. The calibrations of the x-ray scattering instruments were done by using a silver behenate standard and a silicon powder standard. All the 2D scattering patterns were integrated into 1D plots of scattering intensity (*I*) versus *q*, where *q* = 4πsin(θ)/λ and the scattering angle is 2θ.

### Transmission electron microscopy

The preparation of TEM samples is illustrated in fig. S5. Cross-linked H_1_ samples were immersed into a 0.1 wt % KI aqueous solution for 1 hour to enhance atomic number contrast. The stained sample was rinsed using water and completely dried before sectioning. The stained, cross-linked samples were then embedded into an epoxy specified for microtoming. The epoxy resin was cured at 50°C for 12 hours to ensure the required rigidity for sectioning. Samples were microtomed at room temperature with a diamond knife mounted on a Leica EM UC7 ultramicrotome. The thickness of the sections was set to 150 nm with the microtoming instrument. Sectioned samples were then transferred to a TEM grid and characterized using an FEI Tecnai Osiris TEM with an accelerating voltage of 200 kV.

### Atomic force microscopy

AFM studies on the surface morphology of the H_1_ membranes were performed using the tapping mode of a Bruker Dimension FastScan AFM instrument.

### Rheological characterization

Mechanical characterization of the cross-linked H_1_ material was performed on an ARES-G2 rheometer (TA Instruments) in the dynamic mode to determine the shear modulus of the system, using an 8-mm parallel plate with a gap height of ~2 mm.

### Scanning electron microscopy

SEM imaging on cross sections of the H_1_ membranes on supports was conducted using a Hitachi SU-70 instrument with an accelerating voltage of 5 kV.

### UV-visible spectroscopy

UV-visible (UV-Vis) spectra were recorded in transmission mode using a dual-beam configuration on a Cary 300 spectrometer. Dye rejection was quantified by UV-Vis spectrophotometry of permeate solutions (diluted as necessary) compared with UV-Vis absorbances of calibrated dye standard solutions at the characteristic peak absorbance wavelengths of the solutes.

### Fabrication of H_1_/PAN membranes

PAN ultrafiltration membranes with a rejection size of 400 kDa were obtained from Sterlitech Corporation, and the item number was specified as YMPX3001 (Synder Flat Sheet Membrane). PAN membranes were used in this study as mechanical supports for the H_1_ active membranes. The procedure for the membrane fabrication is briefly illustrated in Fig. 3A. Homogenized H_1_ gel contained in a centrifugal tube was centrifuged at a speed of 14 × 10^3^ rpm for 40 min to completely eliminate bubbles trapped in the gel before use. The degassed H_1_ gel (~5 to 10 mg) was quickly placed on top of a smooth Mylar film (slightly stretched to ensure smoothness), followed by covering by a PAN membrane, with the active PAN layer facing the H_1_ gel. The Mylar/H_1_/PAN construct was then sandwiched by two glass plates. Appropriate pressure was applied on the sandwiched construct for 5 min to ensure spreading of the gel on the PAN support. After that, the glass plates were removed and the gel covered by the Mylar film was exposed to a focused spot UV beam for 10 min (100-W Sunspot SM spot curing system at a distance of ∼2.5 cm) in an N_2_ atmosphere. The Mylar film was then carefully peeled off from the cross-linked H_1_ membrane.

### Filtration testing, static adsorption, and relevant example calculations

The hydraulic permeability and dye rejection quantification procedures were identical for the H_1_/PAN membranes and the control PAN support. Roughly square coupons (approximately 2 cm by 2 cm) were installed into a 50-ml EMD Millipore Amicon (UFSC05001) stirred cell atop of a 4.5-cm-diameter piece of polyester macroporous support (Sterlitech). The surface of the membrane was then covered with a customized circular stainless steel mesh. The setup is shown in fig. S8. Within the filtration cell, the active testing area of the membrane coupon was a circular area with a diameter of 1.1 cm, corresponding to an effective surface area of 0.95 cm^2^. After loading the feed solution into the test cell chamber, compressed nitrogen gas was used to pressurize the test cell to pressures ranging from 0.5 to 80 psi. Permeate was collected in glass vials sealed with parafilm to prevent solvent evaporation.

During the rejection experiments, a constant pressure of 80 psi was maintained, and the cell was stirred at 400 rpm to reduce the concentration polarization. At least 1 ml of permeate was collected for each solute rejection experiment. After completion of tests with one dye and before testing the coupon with the next dye, the stirred cells and the membranes were rinsed with deionized (DI) water thoroughly, followed by filtering at least 3 ml of DI water through the coupon to rinse any residual solutes. To prepare the feed solution for single solute rejection experiments, AB, CV, MB, vitamin B12 (VB12), and lysozyme at a concentration of 0.5 g liter^−1^ were dissolved in DI water. The solution pH for lysozyme solution was adjusted by dissolving the NaOH pellets within until the isoelectric point at pH 11.35 was achieved, as monitored by using an Accumet AB15 pH meter (Fisher Scientific) coupled with pH test strips. Meanwhile, riboflavin (VB2) was dissolved in DI water at a concentration of 0.05 g liter^−1^. In the salt rejection experiment, CoCl_2_ was dissolved in DI water at a concentration of 100 mM. To prepare the feed solution for competitive solute rejection experiments, mixture of solutes was dissolved in DI water. Specifically, the VB2 and CV solution was prepared with an equal solute concentration of 0.05 g liter^−1^, and the CoCl_2_ and CV solution was prepared at a concentration of 100 mM and 0.5 g liter^−1^, respectively.

The free-standing H_1_ thin films used for static solute adsorption experiment were prepared in a similar manner as the H_1_ PAN composite, with minor modifications. Specifically, glass slides with sacrificial polymeric coatings were used to sandwich the film during pressing. Two microscope slides (precleaned; Fisher Scientific) were spin-coated (2000 rpm, 1 min) with aqueous polymer solutions, the first slide with 1 wt % dextran (*M*_n_ ~ 70 kg mol^−1^) and the second slide with 1 wt % chitosan (medium molecular weight) solutions, and were subsequently baked in a convection oven at 85°C for 2 hours. Measured amounts of H_1_ gel (~10 mg) were sandwiched between a chitosan-coated slide and a dextran-coated slide. Upon the completion of photoinitiated cross-linking, the glass slide–sandwiched H_1_ thin film was plunged in a DI water bath for a few hours until the dextran coating was fully dissolved to allow removal of the top slide. Following this, the H_1_ gel adhered to the bottom chitosan-coated slide was immersed in a 3 wt % acetic acid bath for 24 hours, causing the dissolution of the chitosan coating and leading to a free-standing floating cross-linked thin film of the H_1_ gel. Subsequently, pieces of free-standing thin films were then immersed in targeted solute solutions for 48 hours with a packing ratio of ~0.2 g of membrane per liter of solution. In the end, the concentrations of the solutions with H_1_ free-standing thin films were analyzed and compared with the reference stock solutions.

The hydraulic permeability was quantified by measuring the amount of time required to collect a certain volume into the collection vial. The following formula was used

$$J=\frac{\left(m_{\text{vial},\text{final}}-m_{\text{vial},\text{init}})\times (1/\rho \right)}{A\times (t_{\text{final}}-t_{\text{final}})\times P}=[\frac{\text{liter}}{m^{2}∙\text{hour}∙\text{bar}}]$$

where *m* is the mass of vial (in grams), ρ is the density of water (in g liter^−1^), *A* is the active membrane testing area (in m^2^), *t* is the time (in hours), and *P* is the gauge pressure (in bar).

A sample calculation is shown below for water permeability of an H_1_ membrane

$$A=\frac{\pi D^{2}}{4}=\frac{\pi {\left[1.1\times 10^{-2}m\right]}^{2}}{4}=9.5\times 10^{-5}m^{2}$$

$$P=80\;\text{psi}\times \frac{1\;\text{bar}}{14.5\;\text{psi}}=5.5\;\text{bar}$$

$$J=\frac{\left(1.75)\times (1/1000\right)}{\left(9.5\times 10^{-5})\times (29)\times (5.5\right)}=0.12\;\text{liters}\;m^{-2}{\text{hour}}^{-1}{\text{bar}}^{-1}$$

UV-Vis spectrophotometry was used for concentration determinations to quantify dye rejection/selectivity performance of the tested membranes. The concentration of the permeate was determined from the linear regression plot from a series of standard concentrations. The dye rejection metric was calculated from UV-Vis spectra as shown here

$$\%\text{Rejection}=100\%\times (1-\frac{c_{\text{permeate}}}{c_{\text{feed}}})$$

where *c*_standard_ is the concentration of standard dye solution (as weight fraction in DI water) and *c*_feed_ is the concentration of feed dye solution (as weight fraction in DI water).

### CFU enumeration assay

*E. coli* (American Type Culture Collection, BW26437) were received from the Yale *E. coli* Genetic Stock Center. Bacteria were grown overnight in Luria-Bertani (LB) broth at 37°C. After incubation, the culture was diluted in a fresh medium and grown until log phase (∼1.5 hours), as evidenced by an optical density of ~0.8 at 600 nm. The bacterial cells were washed three times with sterile saline solutions (0.9 wt % NaCl) before use. CFU enumeration assay was used to evaluate the antimicrobial activity of the H_1_ membranes. The bacterial suspension (10^8^ CFU ml^−1^) was exposed to the membrane surface for 3 hours at room temperature. After discarding the excess bacterial suspension, a 5-ml saline solution was used to rinse unattached cells from the film. The film was then transferred into another 5-ml saline solution and sonicated for 10 min in an ultrasonic bath (26 W liter^−1^; FS60 Ultrasonic Cleaner) to detach bacteria from the film surface. After detachment of cells from the membrane surface, the supernatant was immediately cultured on an LB agar media and incubated overnight at 37°C for CFU enumeration. Bare PAN membranes were also investigated as controls for comparison. Three independent samples were exposed to *E. coli*, and the average value with 1 SD was reported. All results were presented as means ± SD. Statistical differences (*P* < 0.05) between two groups were determined using Student’s *t* test with paired two-tailed distribution.

## Supplementary Material

http://advances.sciencemag.org/cgi/content/full/5/8/eaav9308/DC1

Download PDF

## SUPPLEMENTARY MATERIALS

Supplementary material for this article is available at http://advances.sciencemag.org/cgi/content/full/5/8/eaav9308/DC1 Fig. S1. METDAB/water binary phase diagram as determined by POM and x-ray scattering. Fig. S2. Polymerization of H_1_ mesophases formed by METDAB/water binary systems in the absence of cross-linkers. Fig. S3. Structural characterization of an H_1_ mesophase containing only one cross-linking species in the hydrophobic core of cylindrical micelles before and after UV-initiated cross-linking. Fig. S4. X-ray scattering and POM data showing slight structural changes in the H_1_ gel, the cross-linked H_1_ mesophase, and the swelled polymer. Fig. S5. Schematic illustration for the preparation of TEM samples. Fig. S6. Schematic illustration of the pore dimensions. Fig. S7. SEM images showing the cross sections of the H_1_/PAN composite membranes. Fig. S8. Photos showing the stirred cell used for the nanofiltration test. Fig. S9. The time-dependent solute rejection for H_1_ composites and the static solute adsorption experiment for free-standing H_1_ membranes. Fig. S10. UV-Vis spectrum and photographs demonstrating the competitive solute separation of CV and VB2. Section S1. Calculation of the pore dimensions in an H_1_ membrane
