# Supplementary material for: Precise nanofiltration in a fouling-resistant self-assembled membrane with water-continuous transport pathways
Source: Sci Adv. 2019 Aug 9;5(8):eaav9308. doi: 10.1126/sciadv.aav9308 (PMC6688870; doi:10.1126/sciadv.aav9308)
Supplement: http://advances.sciencemag.org/cgi/content/full/5/8/eaav9308/DC1 [file supp_5_8_eaav9308__index.html]

Science Advances | Science AdvancesAAASSearchScience AdvancesMenu

## Supplementary Materials

**This PDF file includes:**

- Fig. S1. METDAB/water binary phase diagram as determined by POM and x-ray scattering.
- Fig. S2. Polymerization of H1 mesophases formed by METDAB/water binary systems in the absence of cross-linkers.
- Fig. S3. Structural characterization of an H1 mesophase containing only one cross-linking species in the hydrophobic core of cylindrical micelles before and after UV-initiated cross-linking.
- Fig. S4. X-ray scattering and POM data showing slight structural changes in the H1 gel, the cross-linked H1 mesophase, and the swelled polymer.
- Fig. S5. Schematic illustration for the preparation of TEM samples.
- Fig. S6. Schematic illustration of the pore dimensions.
- Fig. S7. SEM images showing the cross sections of the H1/PAN composite membranes.
- Fig. S8. Photos showing the stirred cell used for the nanofiltration test.
- Fig. S9. The time-dependent solute rejection for H1 composites and the static solute adsorption experiment for free-standing H1 membranes.
- Fig. S10. UV-Vis spectrum and photographs demonstrating the competitive solute separation of CV and VB2.
- Section S1. Calculation of the pore dimensions in an H1 membrane

Download PDF

**Files in this Data Supplement:**

- Adobe PDF - aav9308\_SM.pdf
