# Supplementary material for: Precise nanofiltration in a fouling-resistant self-assembled membrane with water-continuous transport pathways
Source: Sci Adv. 2019 Aug 9;5(8):eaav9308. doi: 10.1126/sciadv.aav9308 (PMC6688870; doi:10.1126/sciadv.aav9308)
Supplement: Download PDF [file aav9308_SM.pdf]

Science Advances
AAAS

advances.sciencemag.org/cgi/content/full/5/8/eaav9308/DC1

Supplementary Materials for

Precise nanofiltration in a fouling-resistant self-assembled membrane with water-continuous transport pathways

Xunda Feng, Qaboos Imran, Yizhou Zhang, Lucas Sixdenier, Xinglin Lu, Gilad Kaufman, Uri Gabinet, Kohsuke Kawabata, Menachem Elimelech, Chinedum O. Osuji*

*Corresponding author. Email: cosuji@seas.upenn.edu

Published 9 August 2019, *Sci. Adv.* **5**, eaav9308 (2019)
DOI: 10.1126/sciadv.aav9308

This PDF file includes:

Fig. S1. METDAB/water binary phase diagram as determined by POM and x-ray scattering.
Fig. S2. Polymerization of H_1 mesophases formed by METDAB/water binary systems in the absence of cross-linkers.
Fig. S3. Structural characterization of an H_1 mesophase containing only one cross-linking species in the hydrophobic core of cylindrical micelles before and after UV-initiated cross-linking.
Fig. S4. X-ray scattering and POM data showing slight structural changes in the H_1 gel, the cross-linked H_1 mesophase, and the swelled polymer.
Fig. S5. Schematic illustration for the preparation of TEM samples.
Fig. S6. Schematic illustration of the pore dimensions.
Fig. S7. SEM images showing the cross sections of the H_1/PAN composite membranes.
Fig. S8. Photos showing the stirred cell used for the nanofiltration test.
Fig. S9. The time-dependent solute rejection for H_1 composites and the static solute adsorption experiment for free-standing H_1 membranes.
Fig. S10. UV-Vis spectrum and photographs demonstrating the competitive solute separation of CV and VB2.
Section S1. Calculation of the pore dimensions in an H_1 membrane

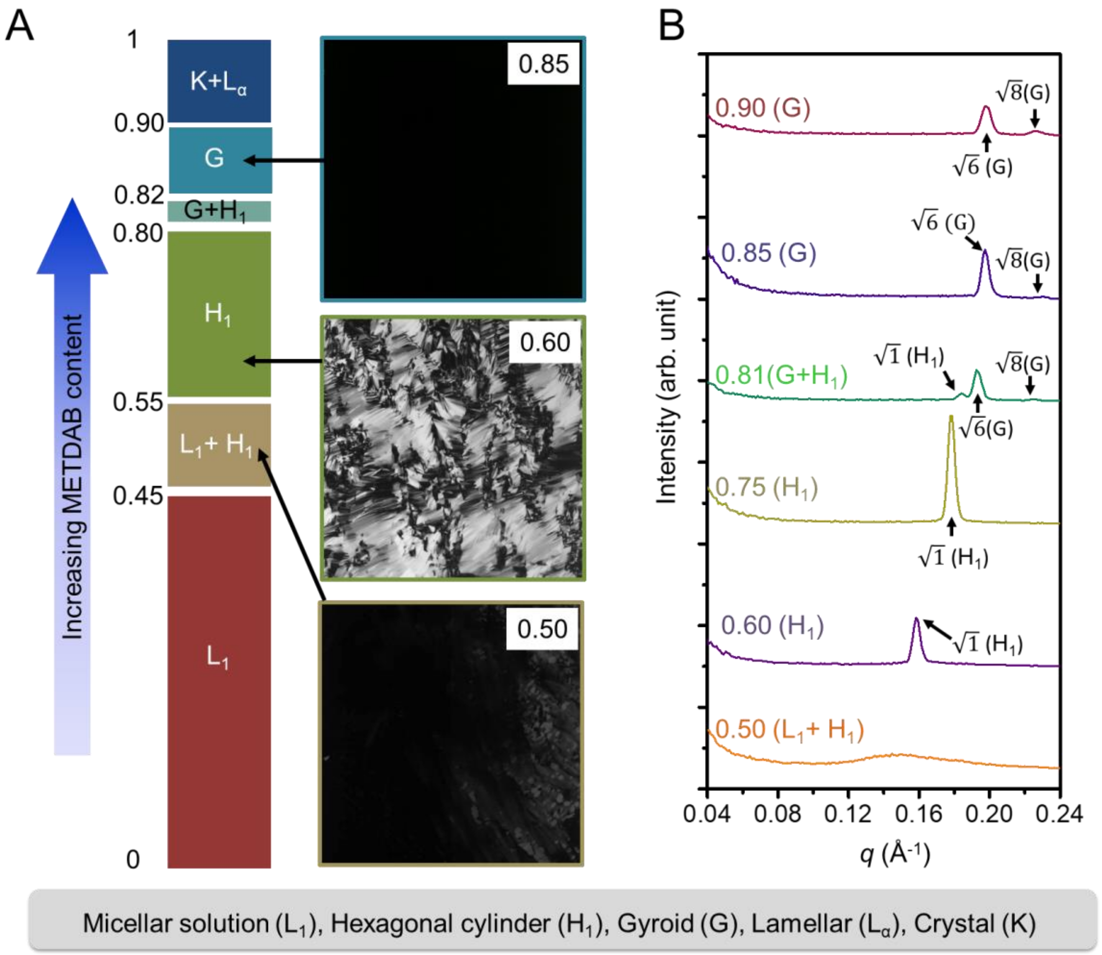

Fig. S1. METDAB/water binary phase diagram as determined by POM and x-ray scattering. The weight concentrations of METDAB are shown in the phase diagram. As the concentration of METDAB was increased, the surfactant/water mixtures followed a phase sequence of micellar solution (L_1), hexagonal cylinder (H_1), gyroid (G), lamellar (L_α), and crystal (K). Selective (A) POM images and (B) X-ray scattering data represent this sequence.

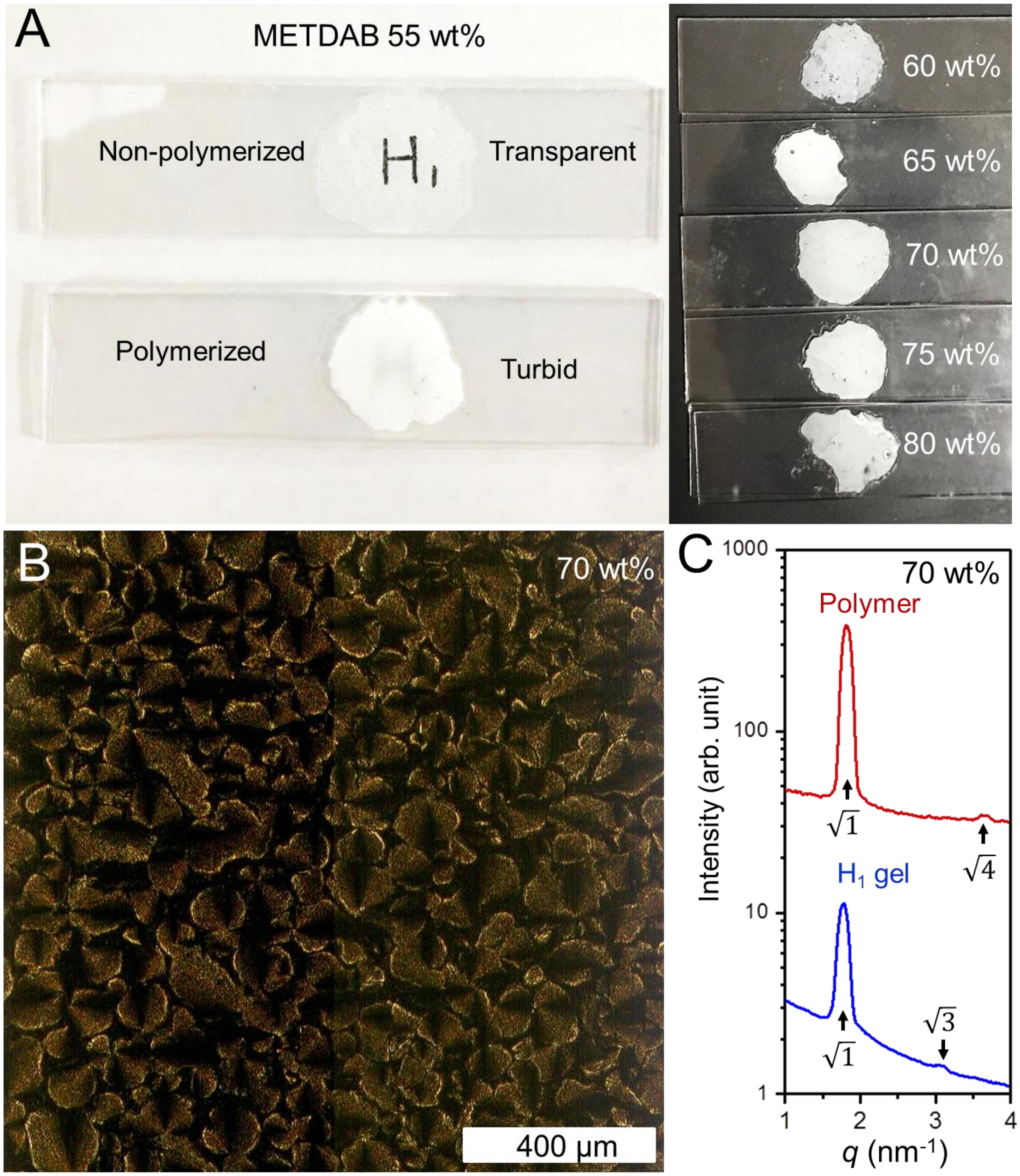

Fig. S2. Polymerization of H_1 mesophases formed by METDAB/water binary systems in the absence of cross-linkers. (A) Photos showing remarkable cloudiness in polymerized H_1 samples with different METDAB contents ranging from 55 to 80 wt%. (B) POM image of the polymerized H_1 mesophase with 70 wt% METDAB shows loss of the typical LC texture. (C) 1-D SAXS data displays that the ratio of peak locations changes from 1: √3 to 1: √4 after polymerization, indicative of disruption of the H_1 morphology. Photo credit: Xunda Feng, Yale University.

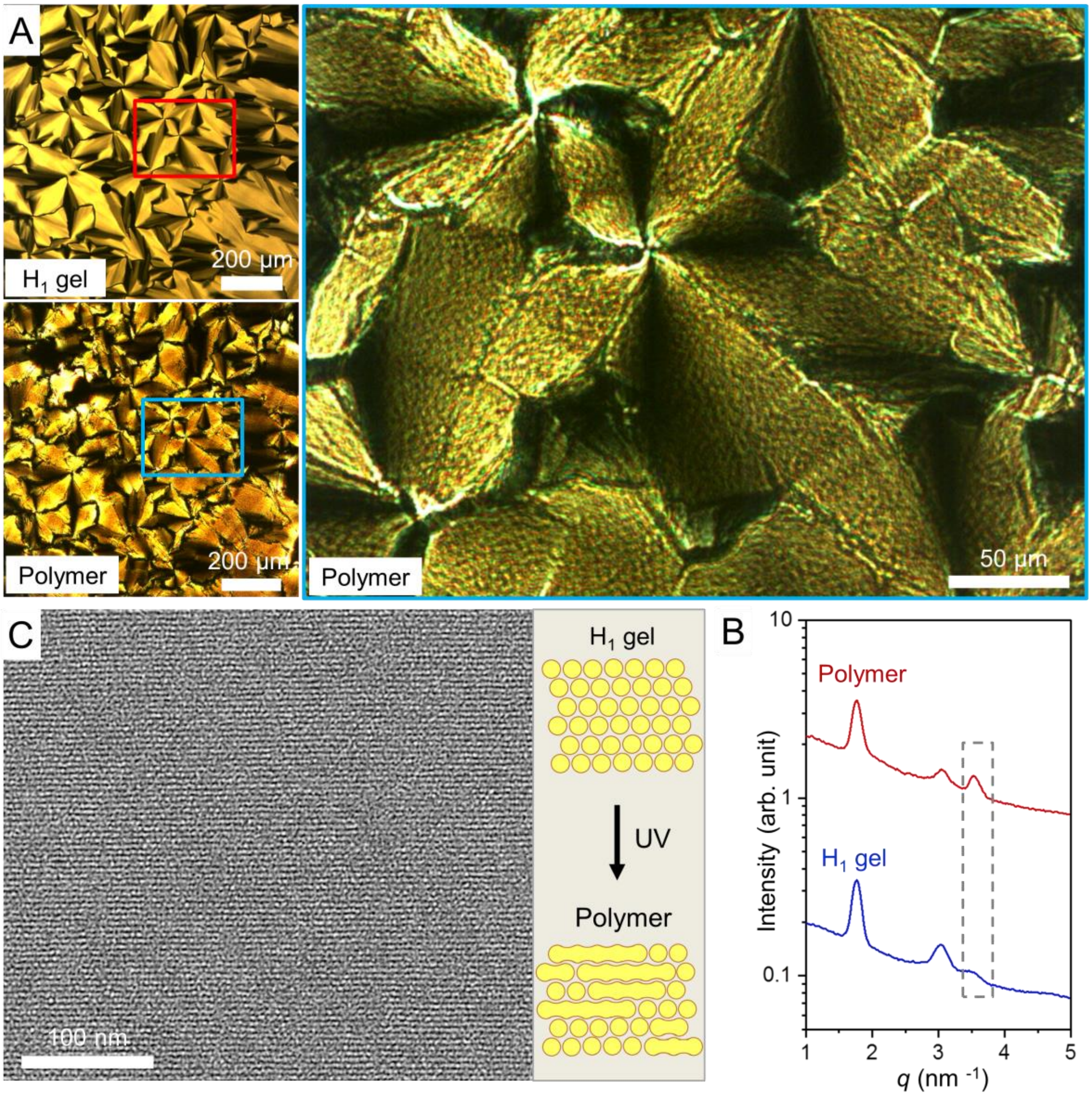

Fig. S3. Structural characterization of an H_1 mesophase containing only one cross-linking species in the hydrophobic core of cylindrical micelles before and after UV-initiated cross-linking. The cross-linker is 1,6-hexanediol dimethacrylate (HDMA). The mesophase contained 70 wt% METDAB, 6 wt% HDMA, and 24 wt% water. (A) Characteristic developable domain texture of the H_1 mesophase observed using POM before crosslinking. After UV induced cross-linking, a superficially similar, but discernably different, birefringent texture was observable in low magnification POM images. A magnified view (right) more clearly shows the emergence of optical inhomogeneities within the developable domains, reflecting disruption of the original H_1 morphology. Rectangles highlight the change of the LC texture induced polymerization. (B) X-ray scattering data showing intact peak locations but an unexpected increase of the intensity of the (200) peak after UV-induced cross-linking. (C) TEM image and schematic, illustrate the tendency of the hexagonal cylinders to transform to lamellar structures after cross-linking.

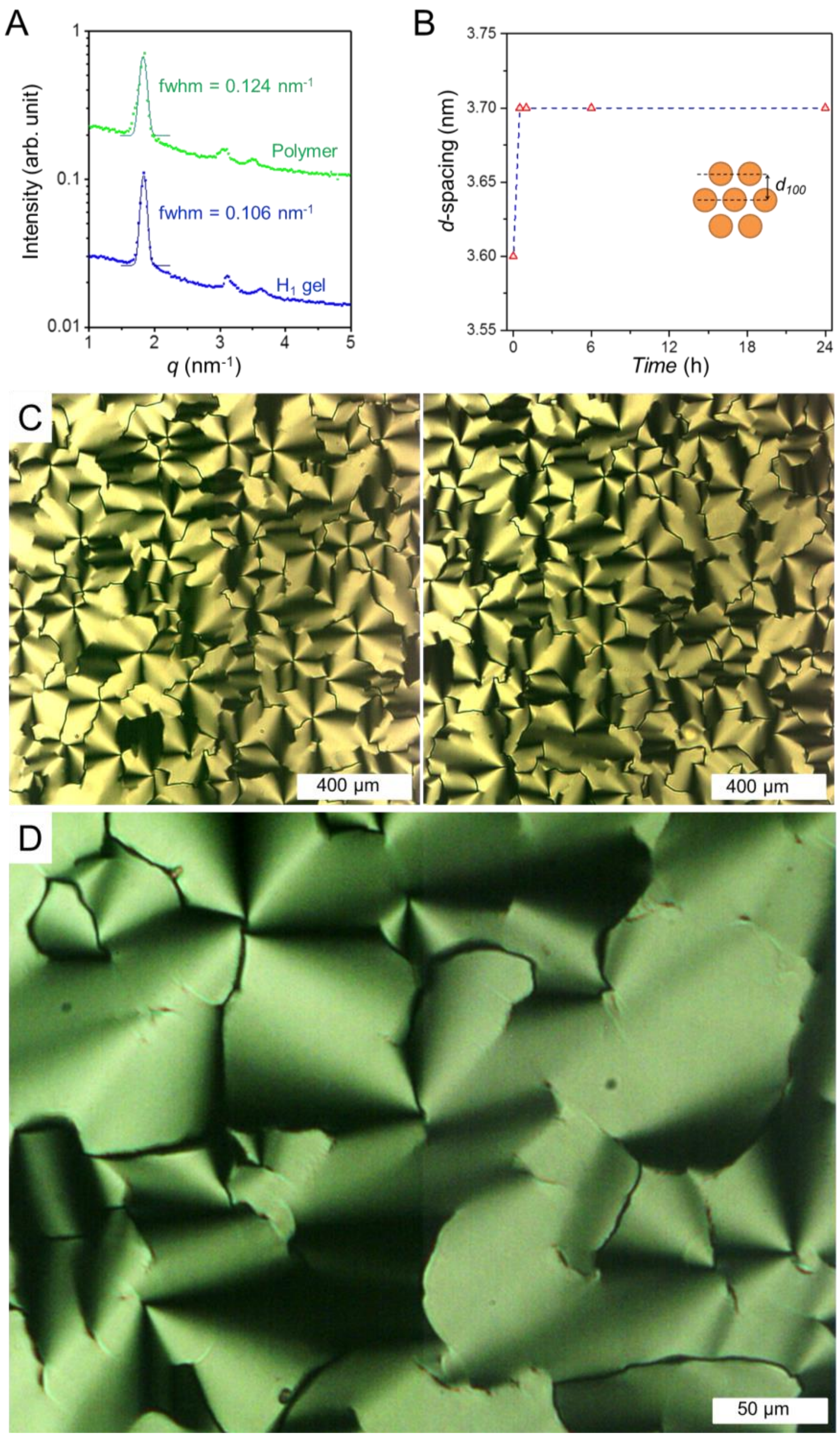

Fig. S4. X-ray scattering and POM data showing slight structural changes in the H_1 gel, the cross-linked H_1 mesophase, and the swelled polymer. (A) Gaussian fits of the (100) SAXS peaks of the H_1 gel and the corresponding polymer to obtain the values of full width at half maximum (fwhm). (B) Time dependent d_{100} spacing of the cross-linked H_1 membrane immersed in water as determined by X-ray scattering. (C) Low-magnification and (D) High-magnification POM images of the crosslinked H_1 mesophase.

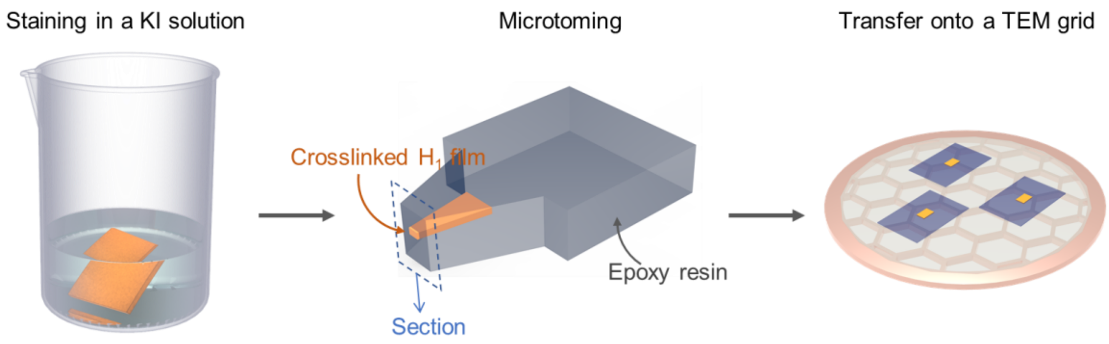

Fig. S5. Schematic illustration for the preparation of TEM samples.

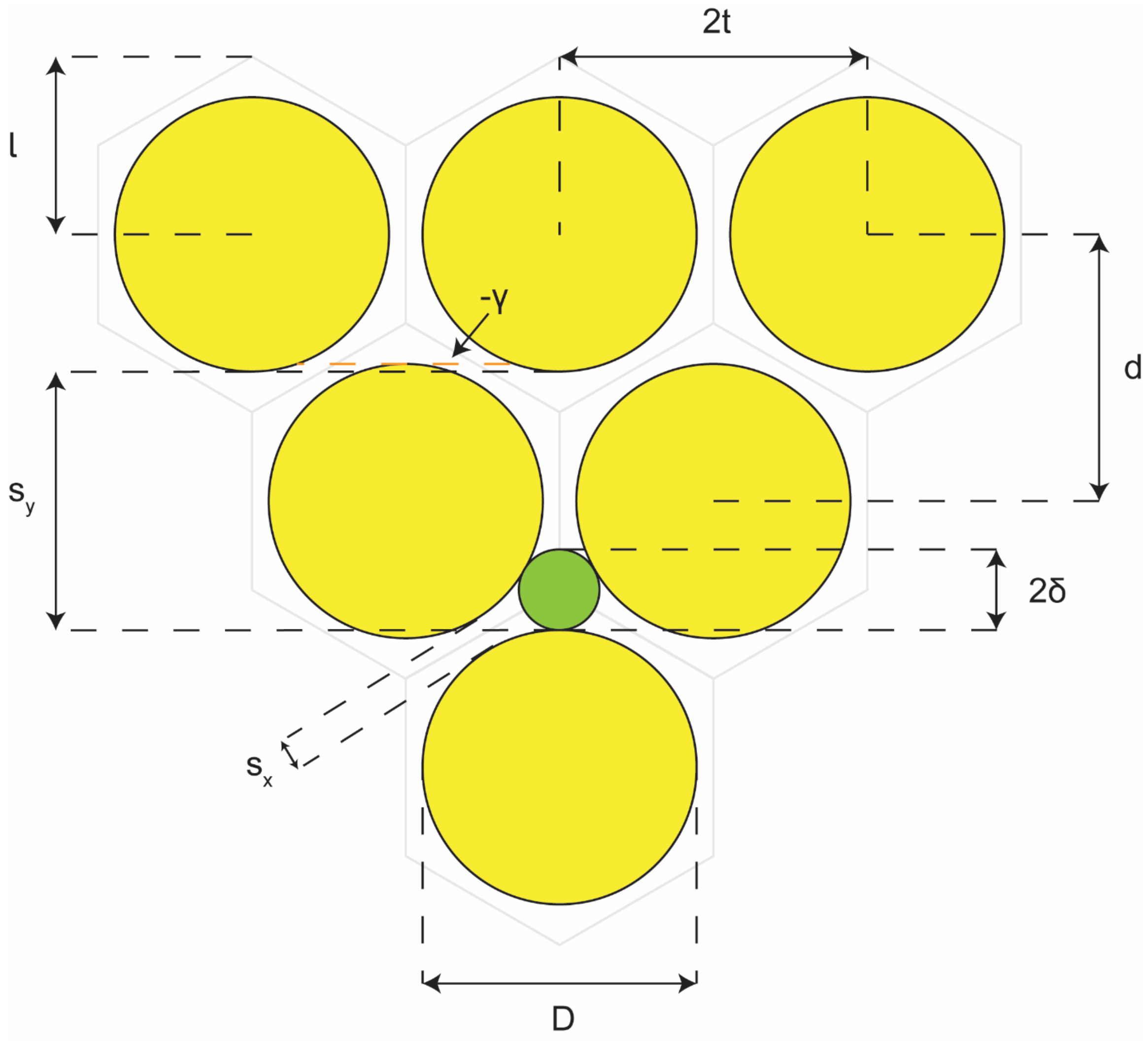

Fig. S6. Schematic illustration of the pore dimensions. The controlling dimensions of the structure are ~ 1.1 nm (2 δ) for transport in parallel and ~ 0.5 nm (S_x) for transport in perpendicular. Calculations are detailed in Section S1.

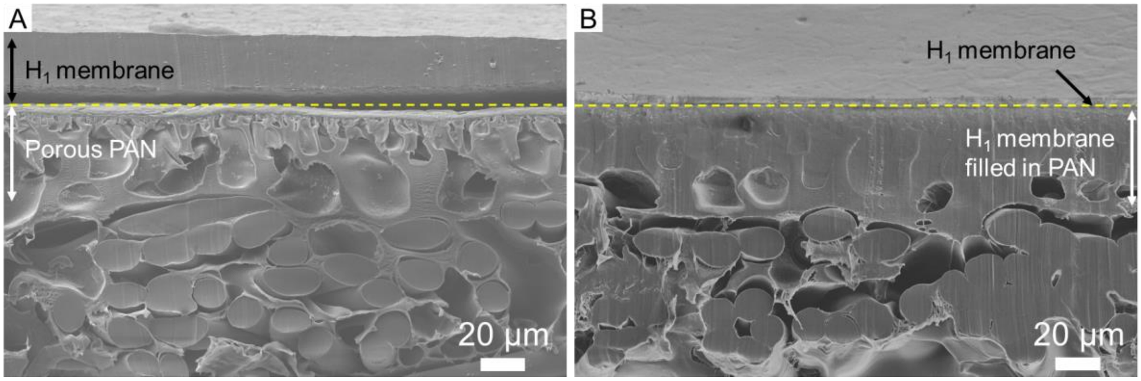

Fig. S7. SEM images showing the cross sections of the H_1/PAN composite membranes. The H_1 gel infiltrates the pores of the PAN support membrane during the pressing stage and the infiltrated H_1 is eventually crosslinked along with the surface H_1 layer.

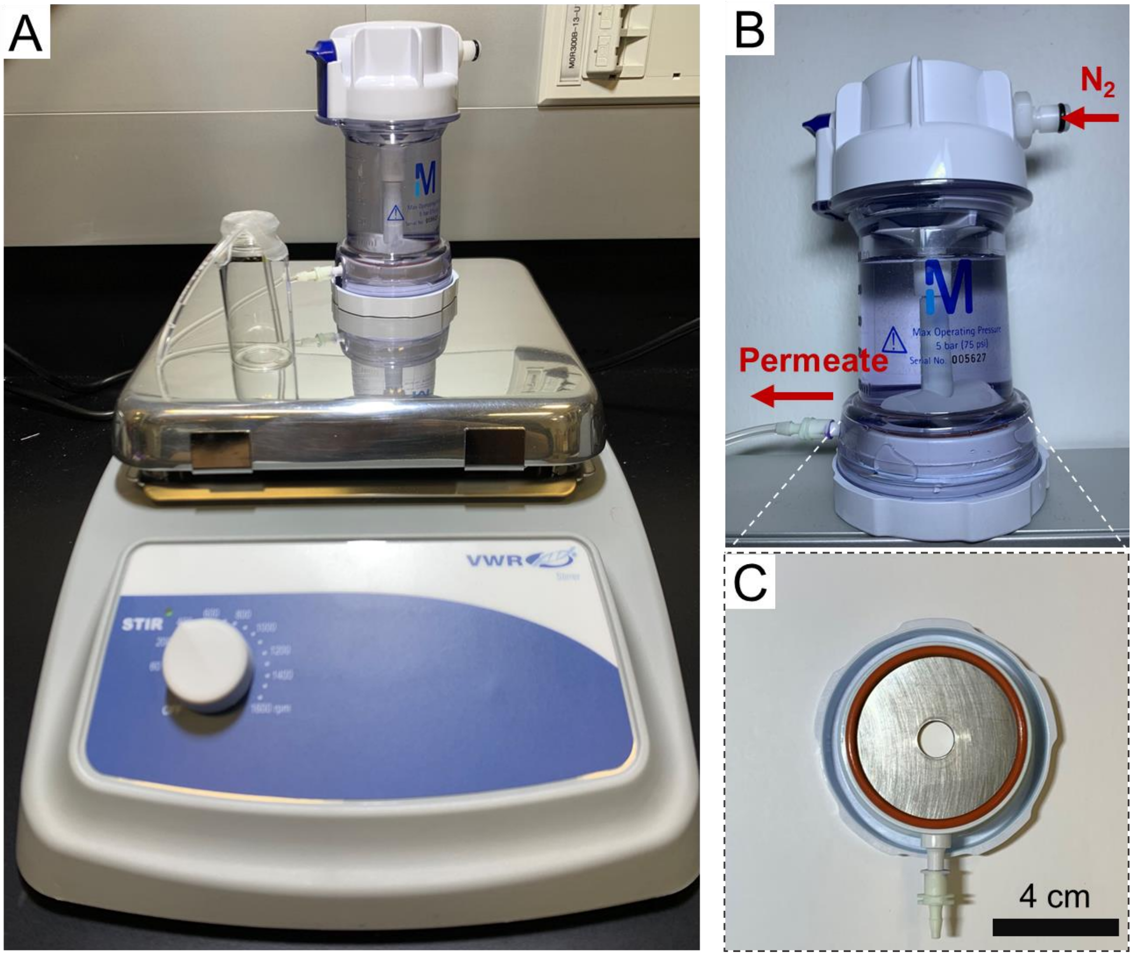

Fig. S8. Photos showing the stirred cell used for the nanofiltration test. (A) 50 mL EMD Millipore Amicon (UFSC05001) stirred cell. A stirring speed of 400 rpm was employed. (B) Photo showing the inlet of the compressed N_2 and the outlet of the permeate. (C) The active testing area of the membrane coupon was a circular area with a diameter of 1.1 cm. Photo credit: Yizhou Zhang, University of Pennsylvania.

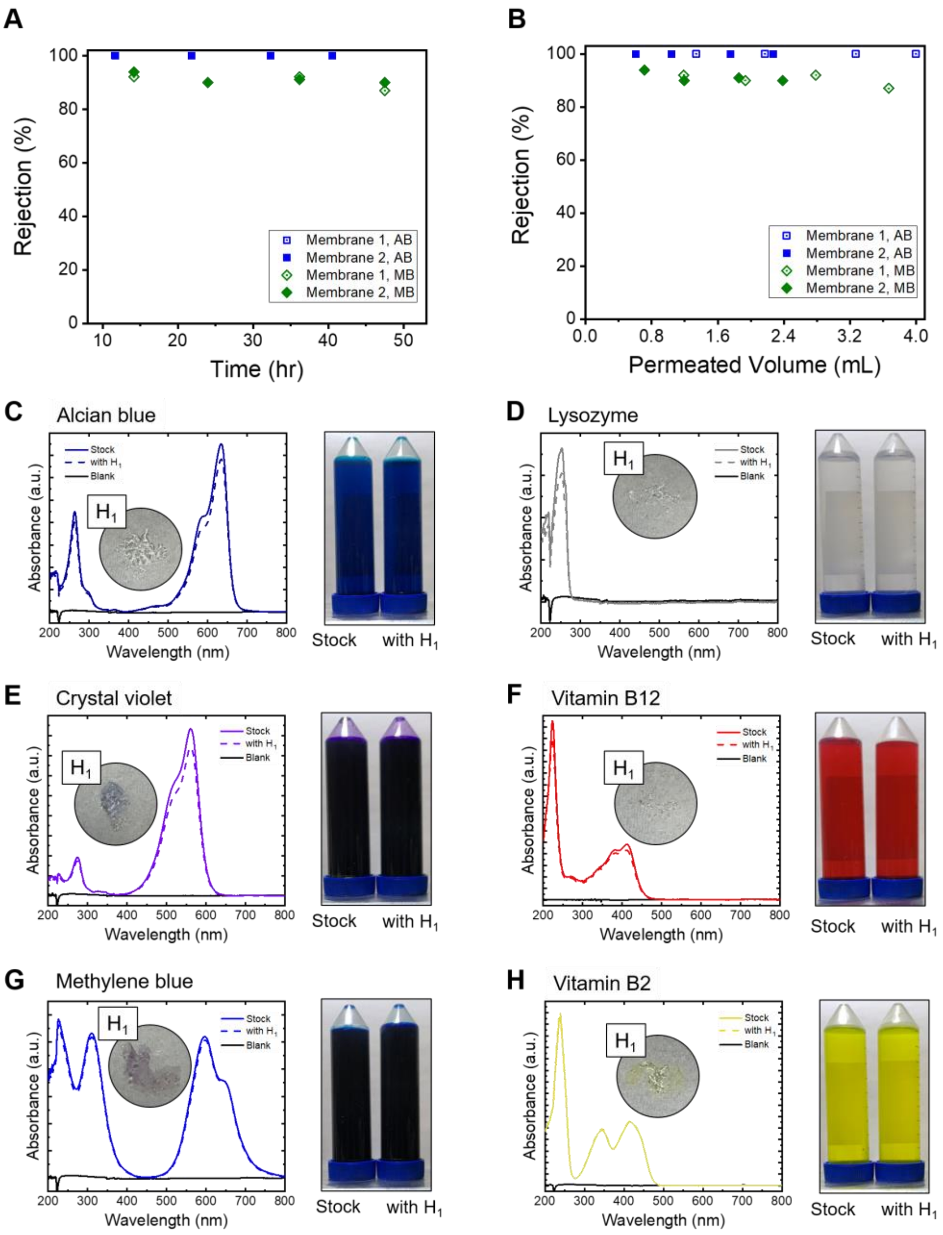

Fig. S9. The time-dependent solute rejection for H_1 composites and the static solute adsorption experiment for free-standing H_1 membranes. The time-dependent solute rejection tests suggest the separation performance of H_1 composite is independent of (A) experiment time and (B) permeated solution volume. UV-Vis spectroscopy was employed to determine the possible adsorption of dye molecules by the membranes. Dyes tested were (C) Alcian blue, (D) lysozyme, (E) crystal violet, (F) vitamin B_{12}, (G) methylene blue and (H) vitamin B_2. The experiment was performed with a same initial solute concentration as utilized during single solute rejection experiment, with a packing ratio of ~0.2 g membrane per L solution. The membranes did not uptake a significant amount of solute during prolonged soaking, suggesting adsorption did not affect the results in single solute rejection experiments. Photo credit: Yizhou Zhang, University of Pennsylvania.

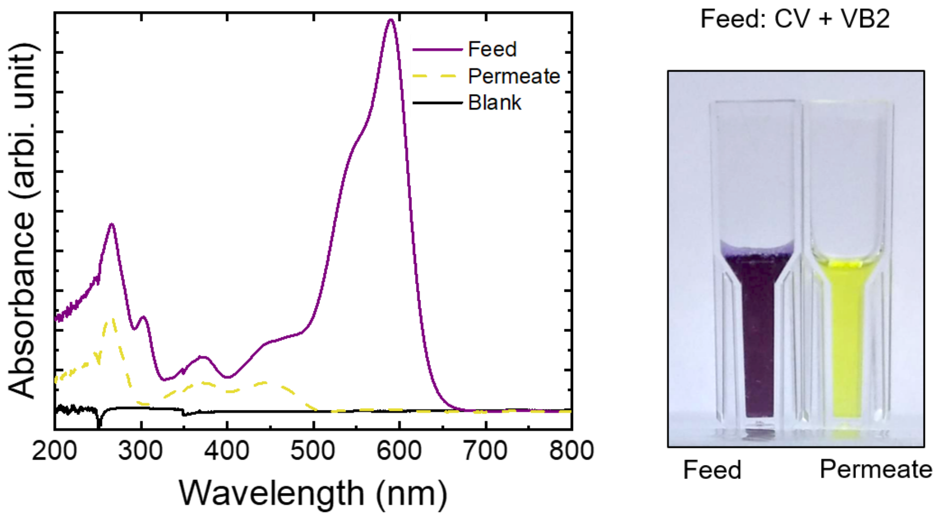

Fig. S10. UV-Vis spectrum and photographs demonstrating the competitive solute separation of CV and VB2. The membrane selectively rejects CV while allowing VB2 to partially permeate through (R ~ 50%). Photo credit: Yizhou Zhang, University of Pennsylvania.

Section S1. Calculation of the pore dimensions in an H_1 membrane

As simply illustrated in fig. S6, the geometry of an H_1 membrane consists of a continuous water transport path and discontinuous cylinders (colored in yellow). The volume fractions of the cylinders ϕ and the water transport path 1-ϕ are assumed to be 0.72 and 0.28, respectively, on the basis of the original composition of the H_1 gel. The distance of the neighboring (100) planes d was determined by X-ray scattering to be 3.6 nm.

$$l = \frac{2d}{3}$$

$$\delta = l - \frac{D}{2}$$

$$\mathrm{t} = \sin(\frac{\pi}{3})l$$

$$2\delta = \mathrm{d}\left[\frac{4}{3} - \left(\frac{8\phi}{\sqrt{3}\pi}\right)^{\frac{1}{2}}\right] = 1.09\ \mathrm{nm}$$

$$S_x = 2t - D = \mathrm{d}\left[\sqrt{\frac{4}{3}} - \left(\frac{8\phi}{\sqrt{3}\pi}\right)^{\frac{1}{2}}\right] = 0.45\ \mathrm{nm}$$

$$S_y = 3l - D = \mathrm{d}\left[2 - \left(\frac{8\phi}{\sqrt{3}\pi}\right)^{\frac{1}{2}}\right] = 3.5\ \mathrm{nm}$$

$$\gamma = \frac{3l}{2} - D = \mathrm{d}\left[1 - \left(\frac{8\phi}{\sqrt{3}\pi}\right)^{\frac{1}{2}}\right] = -0.10\ nm$$
